# Migraine, headache, and mortality in women: a cohort study

**DOI:** 10.1186/s10194-020-01091-9

**Published:** 2020-03-17

**Authors:** Jessica L. Rohmann, Pamela M. Rist, Julie E. Buring, Tobias Kurth

**Affiliations:** 1 Institute of Public Health, Charité – Universitätsmedizin Berlin, Chariteplatz 1, 10117 Berlin, Germany; 2 Division of Preventive Medicine, Department of Medicine, Brigham and Women’s Hospital, Harvard Medical School, Boston, MA USA

**Keywords:** Migraine, Headache, Mortality, Women, Epidemiology

## Abstract

**Background:**

Migraine carries a high global burden, disproportionately affects women, and has been implicated as a risk factor for cardiovascular disease. Migraine with aura has been consistently associated with increased risk of cardiovascular mortality. However, published evidence on relationships between migraine or non-migraine headache and all-cause mortality is inconclusive. Therefore, we aimed to estimate the effect of non-migraine headache and migraine as well as migraine subtypes on all-cause and cause-specific mortality in women.

**Methods:**

In total, 27,844 Women’s Health Study participants, aged 45 years or older at baseline, were followed up for a median of 22.7 years. We included participants who provided information on migraine (past history, migraine without aura, or migraine with aura) or headache status and a blood sample at study start. An endpoints committee of physicians evaluated reports of incident deaths and used medical records to confirm deaths due to cardiovascular, cancer, or female-specific cancer causes. We used multivariable Cox proportional hazards models to estimate the effect of migraine or headache status on both all-cause and cause-specific mortality.

**Results:**

Compared to individuals without any headache, no differences in all-cause mortality for individuals suffering from non-migraine headache or any migraine were observed after adjustment for confounding (HR = 1.01, 95%CI, 0.93–1.10 and HR = 0.96, 95% CI: 0.89–1.04). No differences were observed for the migraine subtypes and all-cause death. Women having the migraine with aura subtype had  a higher mortality due to cardiovascular disease (adjusted HR = 1.64, 95%CI: 1.06–2.54). As an explanation for the lack of overall association with all-cause mortality, we observed slightly protective signals for any cancer and female-specific cancers in this group.

**Conclusions:**

In this large prospective study of women, we found no association between non-migraine headache or migraine and all-cause mortality. Women suffering from migraine with aura had an increased risk of cardiovascular death. Future studies should investigate the reasons for the increased risk of cardiovascular mortality and evaluate whether changes in migraine patterns across the life course have differential effects on mortality.

## Introduction

Migraine is a common chronic, intermittent primary headache disorder affecting 15% to 18% of the general adult population, mainly affecting women [1]. According to the 2016 Global Burden of Disease data, migraine is one of the ten most disabling disorders and is ranked second globally for years of life lived with disability [2].

Migraine can be subdivided into migraine with aura and migraine without aura. The migraine aura is characterized by transient neurological symptoms (often lasting 5–60 min before the migraine pain), mostly affecting the visual field [3]. Individuals with migraine have a higher risk for multiple comorbid conditions, including cardiovascular disease [4–7], and some studies have linked this added risk specifically to the aura subtype [7–9].

We have limited knowledge regarding the association of migraine on all-cause mortality. No overall association was found in a 2011 meta-analysis of five pooled studies [10]. More recently, no association with all-cause mortality was observed in two large cohort studies [11, 12]. A recent Taiwanese study found that people with migraine had lower all-cause mortality than individuals without migraine, though this study did only captured in-hospital mortality and many important sources of confounding may have been omitted by the variable selection process [13].

Given the increased risk for cardiovascular mortality but lack of increased risk on all-cause mortality, we are led to believe there may be differential effects of having migraine in general and of migraine aura subtypes on disease-specific mortality. Specifically, perhaps individuals with active migraine have lower risk for other cause-specific death events, particularly female-specific cancers, as these cancers and migraine are linkable to hormone levels. For these reasons, in the present study, we aimed to estimate the effect of having migraine or non-migraine headache, as well as specific migraine subtypes on all-cause and cause-specific mortality in a large population-based cohort study of women.

## Methods

### Study population

The Women’s Health Study (WHS) began as a large, randomized, placebo-controlled trial (*N* = 39,876) designed to test the effects of aspirin and vitamin E on the primary prevention of cardiovascular disease (CVD) and cancer. The design, methods, and main results of the trial have been described in detail elsewhere [14, 15]. Briefly, at baseline (1992–1995), 39,876 US female health professionals aged 45 years or older without a history of cardiovascular disease, cancer, or other major illnesses were randomized to receive low-dose aspirin (versus placebo) or vitamin E (versus placebo) in a 2-by-2 factorial design. After the trial concluded in 2004, the study continued as a cohort design, and participants continued to be followed on an observational basis.

Women were sent follow-up questionnaires requesting information about demographics, lifestyle, and health information twice during the first year and annually thereafter. Written informed consent was obtained from all participants, and the WHS was approved by the Institutional Review Board at Brigham and Women’s Hospital (clinicaltrials.gov identifier: NCT00000479).

### Inclusion and exclusion criteria

At baseline, a total of 28,345 women provided venous blood samples, of which 27,937 could be assayed for cholesterol levels. Of this group, we excluded participants with missing information on the exposure (migraine or headache status at baseline, *N* = 79). We additionally excluded participants who died after randomization but before the 6-month questionnaire (*N* = 9) or for whom no questionnaire was available (*N* = 5), leaving us with 27,844 women included in the present study.

### Assessment of exposure: migraine and non-migraine headache

The main exposure variable for headache was classified into three categories: (1) no history of migraine or headache, (2) non-migraine headache, or (3) any migraine. Migraine and headache were classified as previously described [16]. Briefly, on the baseline questionnaire, participants were asked whether they had ever experienced migraine headaches and whether they had had migraine headaches in the year prior to baseline. Women responding positively to either of these questions were classified as having “any migraine.” Information about non-migraine headache was asked on the questionnaire disseminated 6 months after baseline, since there was no question about general, non-migraine headache on the baseline questionnaire. We classified participants as having “non-migraine headache” if they reported headache on the 6-month questionnaire but reported no migraine history on the baseline questionnaire. Participants reporting neither were classified as having “no history of migraine or headache.”

To further classify the main exposure variable into migraine subtypes, we used additional information, which was obtained on the baseline questionnaires. For this more detailed secondary categorization, five mutually exclusive headache exposure groups were created: no headache history, non-migraine headache, migraine with aura, migraine without aura, and past history of migraine. In an effort to avoid recall bias, we asked only those participants who reported having migraine within the last year about experiencing aura or any other indication that a migraine attack was coming. We classified women who reported such phenomena as having “migraine with aura”, if not, participants reporting active migraine within the last year were classified as having “migraine without aura.” Women who reported a past history of migraine but no active migraine in the last year prior to baseline were classified as having “past history of migraine.” A previous study in the WHS demonstrated good agreement (more than 87%) between self-reported migraine and self-reported symptoms of the International Classification of Headache Disorders II criteria [17].

### Ascertainment of outcomes: all-cause and disease-specific mortality

For the primary outcome, all reported deaths of all causes were confirmed using information from autopsy reports, death certificates, medical records, and information obtained from next of kin or family members. Disease-specific causes of death due to cardiovascular diseases, any cancers, or female-specific cancers, the secondary outcomes, were confirmed by an Endpoints Committee of physicians using medical records. Female-specific cancers included breast cancer, cervical cancer, endometrial cancer, ovarian cancer, and uterine cancer.

### Statistical analysis

In the present study, we computed person-time from the time of dissemination of the 6-month questionnaire (6 months after initial randomization) to the time of death, loss-to-follow-up, or the administrative censorship date of December 31, 2017.

Cox proportional hazards regression models were used to estimate hazard ratios (HRs) and corresponding 95% confidence intervals (95% CI) for the effects of headache status on all-cause mortality using the group of participants with no history of migraine or headache as the reference category. HRs and 95% CIs for the secondary outcomes of cardiovascular mortality, any cancer mortality, and female-specific cancer mortality were also estimated. No violations of the proportional hazard assumption were observed upon visual inspection of the survival distribution curves for the exposure categories plotted over survival time. As a sensitivity analysis for the secondary outcomes, Fine-Gray subdistribution hazard models were used to account for competing risks. For each model, any deaths due to causes other than the cause of interest were treated as competing events.

We report results from models adjusted for age at randomization as well as multivariable-adjusted models. In our multivariable-adjusted models, we included the following variables measured at baseline identified a priori based on prior knowledge to address potential confounding: age (in years) at randomization, body mass index (BMI) categories based on World Health Organization cut-offs (underweight/normal: < 25, overweight: 25–30, or obese: ≥30 kg/m^2^), total cholesterol in Adult Treatment Panel III [18] categories (< 200, 200–239.9, or ≥ 240 mg/dL), smoking status (never, past or current), alcohol consumption (rarely/never, 1–3 drinks per month, 1–6 drinks per week, or ≥ 1 drink per day), physical activity (rarely/never, < 1 time per week, 1 to 3 times per week, ≥4 times per week), history of diabetes, history of hypertension (self-reported history of hypertension, systolic blood pressure > 140, diastolic blood pressure > 90 mmHg or prescription antihypertensive treatment), family history of myocardial infarction in parent aged < 60 years, and family history of cancer (composite variable of colorectal cancer in parents or siblings at any age, ovarian cancer in mother or sister at any age, or breast cancer in mother or sister aged < 60 years).

Very few women were missing covariate information. All covariates included in the multivariable models had 24 or less missing values except for family history of myocardial infarction (*N* = 460), in which case we created a separate category for women missing this information. For the family history of cancer variable, 858 women were missing information on family history of breast cancer and were imputed to no family history of cancer. For the remaining categories with small numbers of missing values, we imputed the most common or reasonable value. For BMI, we imputed the mean BMI value before categorizing (*N* = 23). For categorical covariates, we imputed the reference category (alcohol consumption, *N* = 6; physical activity, *N* = 10; history of diabetes, *N* = 15; history of hypertension, *N* = 7) or the past user category (smoking, *N* = 24).

We performed an additional post-hoc exploratory analysis to assess associations whether mortality hazards were elevated among those with more frequent migraine attacks. For this analysis, we categorized the exposure into six groups: (1) no history of migraine or headache, (2) non-migraine headache only, (3) past history of migraine, (4) active migraine less than once per month in the year before baseline, (5) active migraine about once per month, and (6) active migraine weekly or daily.

In a further post-hoc sensitivity analysis to address potential residual confounding by socioeconomic status (SES), we created a 10-point SES index based on available income and education information, in accordance with previously published work [19]. We re-ran the previously described multivariable models for the primary outcome, all-cause mortality, with additional adjustment for the SES index using the missing indicator method.

We used SAS statistical software (version 9.4) for all analyses.

## Results

Of the 27,844 participating women in this study, 5128 reported any migraine (1435 migraine with aura, 2175 migraine without aura, and 1518 past history of migraine) and 4025 non-migraine headache at baseline. Women who reported having any migraine at baseline were generally younger, less likely to be current smokers, consumed less alcohol and less frequently reported having diabetes or hypertension (Table 1). We present stratified participant characteristics by all headache and migraine subtypes according to the secondary classification in Additional file 1: Supplementary Table 1.

**Table 1:** Baseline characteristics according to migraine or headache status (*N* = 27,844)

Characteristics at baseline	Migraine or headache status		
	No history	Non-migraine headache	Any migraine
	*N* = 18,691	*N* = 4025	*N* = 5128
Age, mean (SD), years	55.2 (7.3)	53.5 (6.4)	53.6 (6.4)
Body mass index, mean (SD), kg/m^2^	25.8 (4.9)	26.2 (5.2)	26.1 (5.1)
Total cholesterol, median (IQR), mg/dL	208 (184–235)	207 (183–235)	209 (184–236)
Smoking status, %			
Never	50.9	52.7	53.8
Past	37.3	35.8	35.0
Current	11.8	11.4	11.1
Alcohol consumption, %			
Rarely/never	42.7	47.2	47.1
1–3 drinks/month	12.9	13.6	14.4
1–6 drinks/week	32.9	31.2	30.3
≥ 1 drink per day	11.4	8.1	8.2
Vigorous physical activity, %			
Rarely/never	37.2	36.4	38.3
< 1 time/week	18.8	20.8	21.5
1–3 times/week	31.9	33.5	30.0
≥ 4 times/week	12.1	9.2	10.3
Diabetes, %	2.4	3.3	2.0
History of hypertension, %	24.8	25.3	26.2
Family history of:, %			
Myocardial infarction^a^	13.6	15.4	15.3
Colorectal cancer^b^	10.7	9.8	9.6
Ovarian cancer^c^	2.8	2.8	3.3
Breast cancer^d^	6.3	5.8	5.9

Numbers may not add up to 100% because of rounding or missing data. All variables were measured at baseline. Medical history information was self-reported

Abbreviations: *SD* Standard deviation, *IQR* Interquartile range

^a^in parent aged less than 60 years

^b^in mother, father, brother or sister at any age

^c^in mother or sister at any age

^d^in mother or sister aged less than 60 years

During a median of 22.7 years of follow-up (range: 0.02 to 24.1 years), a total of 5012 deaths were confirmed, of which 386 were due to cardiovascular disease and 1224 were due to cancer (of these, 342 female-specific cancer).

In Table 2, we report the age- and multivariable-adjusted model results for the associations between headache or migraine status and all-cause and cause-specific mortality. In the adjusted models, the estimated HRs and corresponding 95% CIs for all-cause mortality were 1.01 (0.93–1.10) for the “non-migraine headache” group and 0.96 (0.89–1.04) for the “any migraine” group compared to the “no history” reference group, illustrating a robustly null relationship.

**Table 2:** Age- and multivariable-adjusted hazard ratios for all-cause and disease-specific mortality according to migraine or headache status (*N* = 27,844)

Migraine or headache status:	No history, *(N = 18,691)*	Non-migraine headache, *(N = 4025)*	Any migraine, *(N = 5128)*
	HR -ref-	HR (95% CI)	HR (95% CI)
**All-cause death,*N***	*3614*	*641*	*757*
Age-adjusted^a^	1 (ref)	1.04 (0.96–1.14)	0.95 (0.87–1.02)
Multivariable-adjusted^b^	1 (ref)	1.01 (0.93–1.10)	0.96 (0.89–1.04)
**Cardiovascular death,*N***	*281*	*46*	*59*
Age-adjusted^a^	1 (ref)	1.08 (0.79–1.48)	1.05 (0.79–1.40)
Multivariable-adjusted^b^	1 (ref)	0.99 (0.72–1.36)	1.07 (0.81–1.42)
**Cancer death,*N***	*861*	*164*	*199*
Age-adjusted^a^	1 (ref)	1.01 (0.86–1.20)	0.96 (0.82–1.12)
Multivariable-adjusted^b^	1 (ref)	1.02 (0.86–1.21)	0.98 (0.84–1.15)
**Female-specific cancer death**^c^, ***N***	*248*	*41*	*53*
Age-adjusted^a^	1 (ref)	0.85 (0.61–1.19)	0.86 (0.64–1.16)
Multivariable-adjusted^b^	1 (ref)	0.87 (0.62–1.21)	0.88 (0.65–1.19)

Abbreviations: *HR* Hazard ratio, *CI* Confidence interval, *ref*, reference category

^a^_,_Adjusted for age at baseline

^b^Adjusted for the following variables measured at baseline: age, BMI, total cholesterol, smoking, alcohol use, physical activity, diabetes, hypertension, family history of myocardial infarction, and family history of cancer (categorizations described in detail in the Methods)

^c^Includes breast cancer, cervical cancer, endometrial cancer, ovarian cancer, and uterine cancer

In secondary analyses, we investigated the association between migraine and cause-specific mortality from any cancer, cardiovascular disease, or female-specific cancers. Among those with non-migraine headache, no meaningful increase in mortality rate was observed for cardiovascular mortality (HR_adj_ = 0.99, 0.72–1.36), any cancer death (HR_adj_ = 1.02, 0.86–1.21) or female-specific cancer mortality (HR_adj_ = 0.87, 0.62–1.21). No relationship was observed for the “any migraine” group with the outcomes cardiovascular mortality (HR_adj_ = 1.07, 0.81–1.42), any cancer death (HR_adj_ = 0.98, 0.84–1.15), or female-specific cancer mortality (HR_adj_ = 0.88, 0.65–1.19). We used the Fine-Gray subdistribution hazard models to account for competing risks and obtained similar results (Additional file 1: Supplementary Table 2).

For the more detailed exposure categorization including the migraine subtypes, the HRs (95% CIs) for the primary outcome of all-cause mortality ranged from 0.91 (0.80–1.03) for the group “migraine without aura” to 1.01 (0.93–1.10) for the “non-migraine headache” group in the multivariable-adjusted models with "no history" as the reference category (Table 3).

**Table 3:** Age- and multivariable-adjusted hazard ratios for all-cause and disease-specific mortality according to migraine subtypes or non-migraine headache status (*N* = 27,844)

Migraine or headache status:	No history, *(N = 18,691)*	Non-migraine headache, *(N = 4025)*	Migraine with aura, *(N = 1435)*	Migraine without aura, *(N = 2175)*	Past history of migraine, *(N = 1518)*
	HR -ref-	HR (95% CI)	HR (95% CI)	HR (95% CI)	HR (95% CI)
**All-cause death,*N***	*3614*	*641*	*198*	*253*	*306*
Age-adjusted^a^	1 (ref)	1.04 (0.96–1.13)	0.96 (0.83–1.11)	0.86 (0.76–0.98)	1.02 (0.91–1.15)
MV-adjusted^b^	1 (ref)	1.01 (0.93–1.10)	0.99 (0.85–1.14)	0.91 (0.80–1.03)	1.00 (0.88–1.12)
**Cardiovascular death,*N***	*281*	*46*	*22*	*12*	*25*
Age-adjusted^a^	1 (ref)	1.08 (0.79–1.47)	1.55 (1.00–2.39)	0.64 (0.36–1.14)	1.08 (0.72–1.63)
MV-adjusted^b^	1 (ref)	0.99 (0.72–1.35)	1.64 (1.06–2.54)	0.68 (0.38–1.22)	1.03 (0.68–1.55)
**Cancer death,*N***	*861*	*164*	*48*	*85*	*66*
Age-adjusted^a^	1 (ref)	1.01 (0.86–1.20)	0.86 (0.64–1.15)	1.04 (0.83–1.30)	0.94 (0.73–1.20)
MV-adjusted^b^	1 (ref)	1.02 (0.86–1.21)	0.89 (0.67–1.19)	1.09 (0.87–1.37)	0.93 (0.72–1.19)
**Female-specific cancer**^**c**^**death,*N***	*248*	*41*	*12*	*26*	*15*
Age-adjusted^a^	1 (ref)	0.86 (0.61–1.19)	0.72 (0.40–1.29)	1.05 (0.70–1.58)	0.74 (0.44–1.25)
MV-adjusted^b^	1 (ref)	0.87 (0.62–1.21)	0.74 (0.41–1.32)	1.08 (0.72–1.62)	0.75 (0.45–1.27)

Abbreviations: *HR* Hazard ratio, *CI* Confidence interval, *ref*, reference category; MV-adjusted, multivariable-adjusted

^a^Adjusted for age at baseline

^b^Adjusted for the following variables measured at baseline: age, BMI, total cholesterol, smoking, alcohol use, physical activity, diabetes, hypertension, family history of myocardial infarction, and family history of cancer

^c^Includes breast cancer, cervical cancer, endometrial cancer, ovarian cancer, and uterine cancer

In the cause-specific mortality analyses (Table 3), having migraine with aura resulted in a significantly increased hazard for cardiovascular mortality compared to the reference group of women without any headache or migraine (HR = 1.64, 95% CI: 1.06–2.54). No other headache status group had significant effects on cardiovascular death. We observed slightly protective effects of having migraine with aura on any cancer and female-specific cancers (HR = 0.89, 95% CI 0.67–1.19 and HR = 0.74, 95% CI: 0.41–1.32), which likely explains, at least in part, the lack of a higher all-cause mortality risk in this group. For the detailed migraine subtype classification, the Fine-Gray subdistribution hazard models yielded similar results (Additional file 1: Supplementary Table 3).

We observed similar results for the complete case analysis as we did for our main analysis (results not shown). In the post-hoc exploratory analysis, we did not observe an association between increasing migraine frequency and increased risk of mortality (Table 4).

**Table 4:** Age- and multivariable-adjusted hazard ratios for all-cause mortality according to migraine frequency (*N* = 27,844)

Migraine or headache status:	No migraine (***N*** = 18,691)	Non-migraine headache (***N*** = 4025)	Past history of migraine (***N*** = 1518)	Active Migraine < 1/month (***N*** = 2727)	Active migraine at least monthly (***N*** = 703)	Active migraine weekly or daily (***N*** = 180)
	HR -ref-	HR (95% CI)	HR (95% CI)	HR (95% CI)	HR (95% CI)	HR (95% CI)
**All-cause death,*N***	*3614*	*641*	*306*	*356*	*73*	*22*
Age-adjusted^a^	1 (ref)	1.04 (0.96–1.13)	1.02 (0.91–1.15)	0.91 (0.82–1.02)	0.85 (0.68–1.08)	0.87 (0.58–1.33)
Multivariable-adjusted^b^	1 (ref)	1.01 (0.93–1.10)	1.00 (0.89–1.12)	0.94 (0.84–1.05)	0.94 (0.74–1.19)	0.92 (0.61–1.40)

Abbreviations: *HR* Hazard ratio, *CI* Confidence interval, *ref*., reference category

^a^_,_Adjusted for age at baseline

^b^Adjusted for the following variables measured at baseline: age, BMI, total cholesterol, smoking, alcohol use, physical activity, diabetes, hypertension, family history of myocardial infarction, and family history of cancer

In the post-hoc sensitivity analysis to address potential residual confounding by socioeconomic status, additional adjustment for SES-index yielded very similar point estimates to the original multivariable-adjusted models. For the main exposure categorization, the estimated HRs and corresponding 95% CIs for all-cause mortality were 1.01 (0.92–1.09) for the “non-migraine headache” group and 0.95 (0.88–1.03) for the “any migraine” group compared to the “no history” reference group. For the detailed secondary categorization including migraine subtypes, the HRs (95% CIs) for the primary outcome of all-cause mortality were 1.01 (0.92–1.09) for the “non-migraine headache” group, 0.98 (0.85–1.13) for the “migraine with aura” group, 0.90 (0.79–1.03) for the group “migraine without aura”, and 0.98 (0.87–1.10) for the “past history of migraine” group.

## Discussion

In this large, prospective cohort of women, we found no association between non-migraine headache or any migraine at baseline with all-cause mortality during follow-up. Furthermore, having migraine with aura, migraine without aura, or a past history of migraine were not found to be associated with all-cause death. This lack of association was apparent across all migraine frequency groups. Women with migraine with aura had increased hazard for cardiovascular disease-specific mortality compared to those with no history of headache. There was a suggestion that women with migraine with aura had a lower risk of cancer mortality, which was most evident for female-specific cancer mortality.

### Comparison with other studies

Migraine and non-migraine headache have not been consistently linked with an increased risk of all-cause mortality [10–12]. Some studies even suggested a decreased risk [20]. Migraine, particularly migraine with aura, has been linked with increased risk of cardiovascular disease in prior reports from several individual studies [5, 6, 8, 9, 21] and meta-analyses [4, 7].

Links between migraine and cancer have been assessed in a few studies; these are of interest due to proposed mechanisms of hormonal involvement for both migraine and cancer, particularly for breast cancer [20, 22–25], and ovarian cancer [26]. However, none of these studies evaluated cancer-specific death as an outcome. To the best of our knowledge, no other study has aimed to estimate the effect of migraine on cancer-specific mortality.

Potential biological mechanisms that increase cardiovascular mortality among women with migraine with aura are currently unknown. Potential mechanisms that have been proposed for the increased risk of cardiovascular disease include endovascular dysfunctions, inflammatory processes [27], genetic susceptibility [28, 29], and mechanisms leading to both migraine with aura and cardiovascular disease [30–32]. Potential mechanisms linking migraine and cancer have been mainly proposed via hormone levels [20]. However, as there is a lack of robust evidence linking migraine and cancer, further speculative discussion about such a potential link is currently not warranted.

### Strengths and limitations

Strengths of our study include the large number of women with migraine or non-migraine headache, long follow-up period, homogeneous composition of the cohort, which may reduce confounding of variables related to access to medical care, confirmed causes of death after medical record review or existing death certificate information, information on a number of potential confounding factors, and sensitivity analyses taking potential competing risk of death by other causes into account.

Several limitations should also be considered when interpreting our results. First, information on migraine, migraine characteristics, non-migraine headache, and included covariates were self-reported, and misclassification is possible. We cannot absolutely rule out that some of the participants classified as having migraine with aura actually experienced other events such as transient ischemic attacks, which were misdiagnosed by healthcare practitioners or by the individual participant themselves, if they did not seek medical care. However, given the usual age of migraine onset compared to other vascular events with some symptomatic overlap, we believe that this would be a rare scenario in our cohort. 

Second, participating women were all healthcare professionals, and results may not be fully generalizable to other female populations and should not be extrapolated to men.

Third, although we are not aware of any major sources of confounding for which we lacked information, we cannot rule out that certain comorbid conditions present at baseline that were not assessed may have impacted risk for migraine status at baseline as well as long-term risk for mortality, introducing some unmeasured confounding. An additional post-hoc sensitivity analysis additionally adjusting our multivariable models for SES index yielded minimal change in the observed effect estimates, suggesting that there was little residual confounding due to SES as measured by the index.

Lastly, we unfortunately had no detailed information on individual trajectories of migraine and non-migraine headache during follow-up and thus could not estimate the influence of patterns and changes in migraine and non-migraine headache over time and risk of mortality.

## Conclusions

In this large prospective study of women, we found no association between non-migraine headache or migraine (regardless of migraine aura status) and all-cause mortality. Women suffering from migraine with aura were found to have an increased hazard for cardiovascular death compared to women with no history of migraine or headache. Future studies should investigate the underlying reasons for the observed increased risk of cardiovascular mortality for this subtype and evaluate whether changes in migraine patterns across the lifecourse may have differential effects on mortality.

## Supplementary information

**Additional file 1: Supplementary Table S1**. Baseline characteristics according to migraine subtypes or non-migraine headache status (*N* = 27,844). **Supplementary Table S2**. Fine-Gray subdistribution age- and multivariable-adjusted hazard ratios for cause-specific mortality accounting for competing risks according to migraine or headache status (*N* = 27,844). **Supplementary Table S3.** Fine-Gray subdistribution age- and multivariable-adjusted hazard ratios for cause-specific mortality accounting for competing risks according to migraine subtypes or non-migraine headache status (*N* = 27,844).

## Data Availability

The data are not publicly available because they contain information that could compromise research participant privacy/consent.

## Abbreviations

BMI: Body Mass Index
CI: Confidence interval
CVD: Cardiovascular disease
HR: Hazard ratio
SES: Socioeconomic status
WHS: Women’s Health Study
