# Supplementary material for: Migraine, headache, and mortality in women: a cohort study
Source: J Headache Pain. 2020 Mar 17;21(1):27. doi: 10.1186/s10194-020-01091-9 (PMC7079482; doi:10.1186/s10194-020-01091-9)
Supplement: Supplementary file 1 — Additional file 1: Supplementary Table S1. Baseline characteristics according to migraine subtypes or non-migraine headache status (N = 27,844). Supplementary Table S2. Fine-Gray subdistribution age- and multivariable-adjusted hazard ratios for cause-specific mortality accounting for competing risks according to migraine or headache status (N = 27,844). Supplementary Table S3. Fine-Gray subdistribution age- and multivariable-adjusted hazard ratios for cause-specific mortality accounting for competing risks according to migraine subtypes or non-migraine headache status (N = 27,844). [file 10194_2020_1091_MOESM1_ESM.pdf]

Supplementary Table 1. Baseline characteristics according to migraine subtypes or non-migraine headache status (N=27,844)

	Migraine or headache status (5 categories)				
**Characteristic at baseline**	No history<br>N = 18,691	Non-migraine headache<br>N = 4,025	Migraine with aura<br>N = 1,435	Migraine without aura<br>N = 2,175	Past history<br>N = 1,518
Age, mean, years	55.2 (7.3)	53.5 (6.4)	53.2 (6.1)	52.6 (5.6)	55.5 (7.4)
Body mass index, mean (SD), kg/m^2	25.8 (4.9)	26.2 (5.2)	25.9 (4.8)	26.3 (5.2)	26.1 (5.1)
Total cholesterol, median (IQR), mg/dL	208 (184-235)	207 (183-235)	208 (184-236)	207 (183-234)	212 (187-240)
Smoking status, %					
Never	50.8	52.7	52.8	56.6	50.8
Past	37.3	35.8	36.6	33.8	35.4
Current	11.8	11.4	10.5	9.6	13.8
Alcohol consumption, %					
Rarely/never	42.7	47.2	48.4	47.5	45.3
1-3 drinks/month	12.9	13.6	13.0	15.5	14.2
1-6 drinks/week	32.9	31.2	30.8	30.1	30.2
≥1 drink per day	11.4	8.1	7.8	6.9	10.3
Vigorous physical activity, %					
Rarely/never	37.2	36.4	37.3	38.3	39.1
<1/week	18.8	20.8	21.1	22.6	20.2
1-3 times/week	31.9	33.5	31.3	29.6	29.2
≥4 times/week	12.1	9.2	10.3	9.5	11.4
Diabetes, %	2.4	3.3	1.7	1.7	2.7
History of hypertension, %	24.8	25.3	23.9	24.1	31.2
Family history of:, %					
Myocardial infarction[a]	13.6	15.4	16.1	14.8	15.2
Colorectal cancer[b]	10.7	9.8	9.8	8.8	10.7
Ovarian cancer[c]	2.8	2.8	3.1	3.0	3.8
Breast cancer[d]	6.3	5.8	6.1	5.6	6.2

Numbers may not add up to 100% because of rounding or missing data. All variables were measured at baseline. Medical history information was self-reported.
Abbreviations: SD, standard deviation; IQR, interquartile range; cig, cigarettes
[a] in parent aged less than 60 years
[b] in mother, father, brother or sister at any age
[c] in mother or sister at any age
[d] in mother or sister aged less than 60 years

Supplementary Table 2. Fine-Gray subdistribution age- and multivariable-adjusted hazard ratios for cause-specific mortality accounting for competing risks according to migraine or headache status (N=27,844).

**Migraine or headache status:**	**No history,** *(N=18,691)*	**Non-migraine headache,** *(N=4,025)*	**Any migraine,** *(N=5,128)*
	HR (95% CI)	***HR (95% CI)***	***HR (95% CI)***
**Cardiovascular death, *N***	*281*	*46*	*59*
Age-adjusted[a]	1 (ref)	1.07 (0.78-1.45)	1.05 (0.79-1.39)
Multivariable-adjusted[b]	1 (ref)	0.98 (0.71-1.34)	1.06 (0.80-1.40)
**Cancer death, *N***	*861*	*164*	*199*
Age-adjusted[a]	1 (ref)	1.01 (0.85-1.19)	0.96 (0.82-1.12)
Multivariable-adjusted[b]	1 (ref)	1.02 (0.86-1.20)	0.98 (0.84-1.15)
**Female-specific cancer death, *N***	*248*	*41*	*53*
Age-adjusted[a]	1 (ref)	0.85 (0.61-1.18)	0.86 (0.64-1.16)
Multivariable-adjusted[b]	1 (ref)	0.87 (0.62-1.21)	0.88 (0.65-1.18)

Abbreviations: HR, hazard ratio; CI, confidence interval; ref, reference category.

Hazard ratios and corresponding 95% confidence intervals were estimated using Fine-Gray subdistribution hazard models. For each model, any deaths due to causes other than the cause of interest were treated as competing events in the models.

[a],Adjusted for age at baseline

[b]Adjusted for the following variables measured at baseline: age, BMI, total cholesterol, smoking, alcohol use, physical activity, diabetes, hypertension, family history of myocardial infarction, and family history of cancer (categorizations described in detail in the Methods).

Supplementary Table 3. Fine-Gray subdistribution age- and multivariable-adjusted hazard ratios for cause-specific mortality accounting for competing risks according to migraine subtypes or non-migraine headache status (N=27,844).

**Migraine or headache status:**	**No history,** *(N=18,691)*	**Non-migraine headache,** *(N=4,025)*	**Migraine with aura,** *(N=1,435)*	**Migraine without aura,** *(N=2,175)*	**Past history of migraine,** *(N=1,518)*
	HR -ref-	***HR (95% CI)***	***HR (95% CI)***	***HR (95% CI)***	***HR (95% CI)***
**Cardiovascular death, *N***	*281*	*46*	*22*	*12*	*25*
Age-adjusted[a]	1 (ref)	1.07 (0.78-1.45)	1.53 (0.99-2.38)	0.65 (0.36-1.15)	1.07 (0.71-1.61)
Multivariable-adjusted[b]	1 (ref)	0.97 (0.71-1.33)	1.63 (1.05-2.53)	0.68 (0.38-1.21)	1.01 (0.67-1.53)
**Cancer death, *N***	*861*	*164*	*48*	*85*	*66*
Age-adjusted[a]	1 (ref)	1.01 (0.85-1.19)	0.84 (0.63-1.14)	1.05 (0.84-1.31)	0.93 (0.73-1.20)
Multivariable-adjusted[b]	1 (ref)	1.02 (0.86-1.21)	0.88 (0.66-1.17)	1.10 (0.88-1.38)	0.93 (0.72-1.20)
**Female-specific cancer death, *N***	*248*	*41*	*12*	*26*	*15*
Age-adjusted[a]	1 (ref)	0.85 (0.61-1.18)	0.71 (0.40-1.28)	1.06 (0.71-1.59)	0.73 (0.44-1.24)
Multivariable-adjusted[b]	1 (ref)	0.87 (0.62-1.21)	0.73 (0.41-1.30)	1.09 (0.72-1.63)	0.75 (0.45-1.26)

Abbreviations: HR, hazard ratio; CI, confidence interval; ref, reference category.

Hazard ratios and corresponding 95% confidence intervals were estimated using Fine-Gray subdistribution hazard models. For each model, any deaths due to causes other than the cause of interest were treated as competing events in the models.

[a],Adjusted for age at baseline

[b]Adjusted for the following variables measured at baseline: age, BMI, total cholesterol, smoking, alcohol use, physical activity, diabetes, hypertension, family history of myocardial infarction, and family history of cancer (categorizations described in detail in the Methods).
